# Exosomes from mesenchymal stem cells expressing miR‐125b inhibit neointimal hyperplasia via myosin IE

**DOI:** 10.1111/jcmm.14060

**Published:** 2018-11-28

**Authors:** Dongqing Wang, Bin Gao, Jianing Yue, Fei Liu, Yifan Liu, Weiguo Fu, Yi Si

**Affiliations:** ^1^ Department of Vascular Surgery Zhongshan Hospital Fudan University Shanghai China; ^2^ Department of Endovascular Surgery the First Affiliated Hospital, Zhengzhou University Henan China; ^3^ Department of Vascular Surgery the Fifth People's Hospital of Shanghai, Fudan University Shanghai China

**Keywords:** exosome, mesenchymal stem cells, miR‐125b, Myo1e, neointimal hyperplasia

## Abstract

Intercellular communication between mesenchymal stem cells (MSCs) and their target cells in the perivascular environment is modulated by exosomes derived from MSCs. However, the potential role of exosome‐mediated microRNA transfer in neointimal hyperplasia remains to be investigated. To evaluate the effects of MSC‐derived exosomes (MSC‐Exo) on neointimal hyperplasia, their effects upon vascular smooth muscle cell (VSMC) growth in vitro and neointimal hyperplasia in vivo were assessed in a model of balloon‐induced vascular injury. Our results showed that MSC‐Exo were internalised by VSMCs and inhibited proliferation and migration in vitro. Further analysis revealed that miR‐125b was enriched in MSC‐Exo, and repressed the expression of myosin 1E (Myo1e) by targeting its 3ʹ untranslated region. Additionally, MSC‐Exo and exosomally transferred miR‐125b repressed Myo1e expression and suppressed VSMC proliferation and migration and neointimal hyperplasia in vivo. In summary, our findings revealed that MSC‐Exo can transfer miR‐125b to VSMCs and inhibit VSMC proliferation and migration in vitro and neointimal hyperplasia in vivo by repressing Myo1e, indicating that miR‐125b may be a therapeutic target in the treatment of vascular diseases.

## 1 INTRODUCTION

Coronary heart disease is a leading cause of morbidity and mortality worldwide.^1^ Treatment options include coronary artery bypass surgery and balloon angioplasty combined with stent implantation^2^; however, the failure rate for treatment is high (8%‐40%) because of restenosis (recurrence of blood vessel narrowing) predominantly caused by intimal hyperplasia.^3^, ^4^, ^5^, ^6^ Intimal hyperplasia is the thickening of a blood vessel wall in response to injury, potentially induced by a surgical procedure. Mesenchymal stem cells (MSCs) have been shown to exert an inhibitory effect on neointimal hyperplasia (the new layer of arterial intima formed particularly on a prosthesis) via rapid re‐endothelialisation.^7^ Another study reported the therapeutic effects of human MSCs by decreasing the inflammatory response to carotid artery ligation.^8^ Exosomes secreted by MSCs have been shown to mediate intercellular communications between these cells and their target cells^9^; however, the role of MSC‐derived exosomes (MSC‐Exo) in neointimal hyperplasia remains to be fully elucidated.

Exosomes, small membraned vesicles (30‐100 nm) that are generated through inward budding of the endosomal membrane, modulate the function of recipient cells by transferring complex biological information, including mRNAs, microRNAs (miRNAs) and soluble proteins into these target cells in a functional form.^10^, ^11^, ^12^ Liang et al showed that transfer of miR‐125a to endothelial cells via MSC‐Exo promoted angiogenesis in these target cells by miR‐125a‐mediated repression of DLL4.^9^ The direct effect of exosomes on target cell function provides a wide range of potential therapeutic applications, including cellular renewal and tissue repair following myocardial ischaemia‐reperfusion,^13^ neurological stroke^14^ and skin wounds,^15^ likely mediated by mechanisms involving exosome‐transferred miRNAs.^16^, ^17^ However, whether exosome‐mediated miRNA transfer plays a role in neointimal hyperplasia remains poorly understood.

In this study, to evaluate MSC‐Exo, and more specifically MSC‐Exo‐derived miRNA transfer on neointimal hyperplasia, we assessed their effects on the proliferation and migration of vascular smooth muscle cells (VSMCs) in vitro and in vivo by the determination of neointima formation following balloon‐induced vascular injury. Previous studies in VSMCs have reported a number of key miRNAs (miR‐21, miR‐143, miR‐145 and miR‐221) that play important roles in proliferation, migration and neointimal thickening.^18^, ^19^, ^20^, ^21^ Another key miRNA, miR‐125b, has been shown to inhibit the process of calcification of VSMCs, a pathology linked to the development of cardiovascular disease,^22^ by inhibiting osteoblastic differentiation and proliferation.^23^, ^24^, ^25^ In our study, the delivery of miR‐125b into VSMCs via MSC‐Exo was investigated along with the potential role of MSC‐Exo‐transferred miR‐125b in neointimal hyperplasia.

Myosin‐1E (Myo1e) is an actin‐dependent molecular motor whose translocation is essential for lamellipodium extension and subsequent cellular motility and cell migration.^26^ Heim et al^27^ reported that Myo1e binds to the FERM domain of focal adhesion kinase (FAK), activating FAK and inducing Y397 phosphorylation. Previous studies revealed that FAK is involved in the regulation of aortic smooth muscle cell motility and growth, and the regulation of smooth muscle cell recruitment during blood vessel morphogenesis.^28^, ^29^, ^30^ The potential role of Myo1e and FAK activation in VSMCs in response to MSC‐Exo‐derived signals was also investigated in this study. Our findings provide insight into the role of MSC‐Exo‐mediated miR‐125b transfer on neointimal hyperplasia following arterial injury, offering potential therapeutic targets for vascular diseases.

## 2 METHODS

### 2.1 Cell culture

Bone marrow MSCs were isolated as previously described.^31^ Briefly, total bone marrow cells were flushed from the femurs and tibias of Sprague‐Dawley rats using culture media. The cells were then cultured in DMEM/F12 (HyClone, Logan, UT, USA), supplemented with 10% FBS (Gibco, Carlsbad, CA, USA) at 37°C in a humidified incubator containing 5% CO_2_. After 48 hours, nonadherent cells were removed by aspirating the media and replacing with fresh media. MSCs of passages 3 to 5 were used for subsequent experiments. MSCs (passage 3) were identified by flow cytometry with antibodies against phycoerythrin (PE)‐CD90, PE‐CD29, fluorescein isothiocyanate (FITC)‐CD44 and FITC‐CD34 (BD Biosciences, Franklin Lakes, NJ, USA). The analysis was performed with a FACSCalibur flow cytometer (BD Biosciences).

Primary rat VSMCs were isolated as previously described^32^ with some modifications. Briefly, the aorta of an adult Sprague‐Dawley rat was excised, cleaned of connective tissue, fat and endothelium and cut into 1‐mm^2^ pieces. The pieces were treated with 1 mL (1 mg/mL) of collagenase II (Sigma‐Aldrich, St. Louis, MO, USA) and 0.125% trypsin for 1 hour at 37°C, followed by centrifugation at 300 *g* for 5 minutes to harvest the cells. Cells were cultured in DMEM supplemented with 10% FBS, and passages 3‐7 were used for subsequent experiments. HEK293 cells were cultured in DMEM containing 10% FBS.

### 2.2 Cell transfection

For miR‐125b overexpression, primary MSCs isolated from rats were transfected with mimic control (NC) or miR‐125b mimic (50 nmol/L; GenePharma, Shanghai, China), and for miR‐125b knockdown, primary MSCs from rats were transfected with an inhibitor control (IC) or miR‐125b inhibitor (100 nmol/L; GenePharma) using Lipofectamine RNAiMAX reagent (Invitrogen, Carlsbad, CA, USA) according to the manufacturer's protocol. The transfected cells were cultured for 48 hours prior to use in subsequent experiments.

For Myo1e overexpression, full‐length rat Myo1e cDNA was inserted into the pcDNA3.1 expression vector (Invitrogen) together with the DNA sequence for an N‐terminal FLAG tag (Myo1e‐FLAG) and cells were transfected as described above. For Myo1e knockdown, vector GV112 plasmids carrying shRNA for Myo1e (target sequence: 5′‐GCATCAACCGAAACTTCATCG‐3′) or control shRNA were purchased from the GeneChem corporation (Shanghai, China). Cells were transfected with either shRNA plasmids for Myo1e or for control as described above.

### 2.3 Isolation and characterisation of exosomes

To isolate the exosomes of MSCs, the cells were cultured in DMEM/F12 containing 10% exosome‐free FBS for 48 hours and the supernatants were collected and centrifuged at 3000 *g* for 15 minutes to remove the cells and cell debris. The exosomes were isolated from the supernatants using ExoQuick‐TC Kit (System Biosciences, Mountain View, CA, USA) according to the manufacturer's instructions. The pelleted exosomes were fixed in 2% paraformaldehyde in PBS, pH 7.4 and the morphology of the exosomes was observed using transmission electron microscopy (TEM) as previously described.^33^ The exosomes were further characterized by Western blot analysis with three exosome‐specific biomarkers: CD9, CD63 and CD81 (Abcam, Cambridge, UK).

### 2.4 Internalisation of DIO‐labelled exosomes into VSMCs

Purified exosomes were labelled with 5 mmol/L of the fluorescent dye DIO (Invitrogen) by incubation for 15 minutes at 37°C. Any remaining free dye was removed by ultracentrifugation at 120 000 *g* for 90 minutes, followed by two washes in PBS with ultracentrifugation.

To analyse exosome uptake by VSMCs, cells were incubated with DIO‐labelled exosomes for 3 hours and then stained with DAPI (Invitrogen). The internalisation of DIO‐labelled exosomes by VSMCs was visualized using an Eclipse TE2000 fluorescence microscope (Nikon, Tokyo, Japan).

### 2.5 Shuttling assays for Cy3‐labelled miRNA

For transfection with Cy3‐labelled miR‐125b mimics, miR‐125b mimics were first labelled with Label IT siRNA Tracker Cy3 kit (Mirus, Madison, WI, USA) according to the manufacturer's instructions. MSCs were transfected with Cy3‐labelled miR‐125b mimics and incubated for 48 hours in medium containing exosome‐free FBS. Then, exosomes were isolated and subsequently incubated with VSMCs for 3 hours. Finally, cells were visualized under a fluorescence microscope as discussed above.

### 2.6 Quantitative reverse‐transcription PCR

Total RNA was extracted from MSCs or MSC‐Exo using the RNeasy mini kit (Qiagen, Hilden, Germany) according to the manufacturer's protocol. Then, cDNAs were synthesized using HiScript Reverse Transcriptase (RNase H; Vazyme Biotech Co. Ltd., Nanjing, China). Quantitative PCR was performed on an ABI7900 Real‐Time PCR System (Applied Biosystems, Foster City, CA, USA) using SYBR Green Master Mix (Vazyme Biotech Co. Ltd.) according to the manufacturer's instructions. The specific primers used in these reactions were as follows: rno‐miR‐125b, forward 5ʹ‐TGCGCTCCCTGAGACCCTAACT‐3ʹ and reverse 5ʹ‐CCAGTGCAGGGTCCGAGGTATT‐3ʹ; U6, forward 5ʹ‐CGCTTCGGCAGCACATATAC‐3ʹ and reverse 5ʹ‐AAATATGGAACGCTTCACGA‐3ʹ; Rat Myo1e, forward 5ʹ‐AAAGCTACCTGGC CTGTGTG‐3ʹ and reverse 5ʹ‐AGGTCTGAGGCGTCTTCTCT‐3ʹ; and β‐actin forward 5ʹ‐CACGATGGAGGGGCCGGACTCATC‐3ʹ and reverse 5ʹ‐TAAAGACCTCTATGCCAACACAGT‐3ʹ. Relative miRNA expression normalized to U6, and relative mRNA expression normalized to β‐actin were determined using the 2^−ΔΔCt^ method.

### 2.7 Western blot analysis

For Western blot analysis of exosome‐derived proteins, the Qproteome Mammalian Protein Prep Kit (Qiagen) was employed to extract the proteins from exosomes. For Western blot analysis of proteins from cell lysates, cells were harvested in RIPA lysis buffer (Beyotime, Haimen, China) containing 1 mmol/L PMSF and protease inhibitor cocktail. A BCA Protein Assay Kit (Pierce, Rockford, IL, USA) was used to quantify the proteins before loading onto a 10% SDS‐PAGE gel. The separated proteins were transferred to PVDF membranes (Millipore Corporation, Bedford, MA, USA), and incubated with specific primary antibodies: Myo1e, anti‐FAK (phospho Y397), anti‐FAK or β‐actin (Abcam), and anti‐Flag (Santa Cruz Biotechnology, Santa Cruz, CA, USA). Then, the membranes were incubated with HRP‐conjugated secondary antibody (Abcam) for 1 hour at room temperature. Signals were detected using enhanced chemiluminescent reagent (Pierce).

### 2.8 Luciferase reporter assay

A synthetic fragment of the rat Myo1e 3′‐UTR containing either wild‐type (WT) or mutant (MUT) miR‐125b binding sites, was inserted into the pRL‐TK‐Report vector (Promega, Madison, WI, USA). HEK293 cells were cotransfected with 200 ng of WT or MUT construct or the vehicle control and the miR‐125b mimic or inhibitor or negative control (50 nmol/L) using Lipofectamine 2000 reagent (Invitrogen) according to the manufacturer's protocol. After 48 hours, the firefly and Renilla luciferase activities were sequentially measured using the Dual‐Glo™ Luciferase Assay system (Promega) and a GloMax microplate luminometer (Promega). The relative luciferase activity was normalized to the Renilla luciferase activity.

### 2.9 Cell proliferation assay

Cell proliferation was determined by an MTT assay. VSMCs were seeded at different densities in 96‐well plates and cultured overnight. The cells were then treated with purified exosomes (200 μg/mL) in the presence or absence of platelet‐derived growth factor (PDGF‐BB; 20 ng/mL; PeProTech, Rocky Hill, NJ, USA). After 48 hours incubation, cell growth was analysed by measuring the optical density at 570 nm using a microplate reader.

### 2.10 Scratch wound healing assay

Vascular smooth muscle cells were seeded in 6‐well plates at a concentration of 2 × 10^5^ cells per well. After incubation with starvation medium for 24 hours, a sterile pipette tip was used to inflict a linear scratch wound in the centre of the cell monolayer. After washing with PBS to remove any cellular debris, cells were stimulated with or without exosomes (200 μg/mL) in the presence or absence of PDGF‐BB (20 ng/mL) for 24 hours, and the wound was monitored under a phase‐contrast microscope (Olympus IX51, Tokyo, Japan), and the percentage of cell closure was calculated by measurements of the scratch width using ImageJ software.

### 2.11 In vivo effects of MSC‐Exo in a rat model of balloon‐induced vascular injury

The balloon‐induced vascular injury model was performed as previously described.^34^ Briefly, Sprague‐Dawley rats weighing 250‐300 g (SLAC Laboratory Animal Co., Ltd, Shanghai, China) were anesthetised with 75 mg/kg pentobarbital and heparinised with 100 U/kg heparin sodium. To induce balloon injury, a 2F Fogarty arterial embolectomy balloon catheter (Edwards Lifesciences, Irvine, CA, USA) was introduced through the left external carotid artery and the artery was distended by the passage of saline three times. After balloon injury, 20 μg of exosomes was injected intravenously every other 2 days for 14 days. The left common carotid artery was harvested for analysis. Immunofluorescence was used to detect the localisation of exosomes in the carotid artery. DIO‐labelled exosomes were injected intravenously into rats following vascular injury. The left common carotid artery was harvested for cryostat sectioning. Carotid artery tissue was embedded in OCT compound and snap frozen for cryostat sectioning (7 μm). Artery tissue sections were then examined under a fluorescence microscope.

All procedures involving animals were approved by the Animal Research Committee of Zhongshan Hospital Fudan University (Reference number: SCXK2009‐0019).

### 2.12 Histomorphometry and immunofluorescence

For histomorphometry and immunofluorescence analysis, excised arteries were fixed in 4% paraformaldehyde for 24 hours, dehydrated, embedded in paraffin and sliced into 5‐μm sections. The sections were then stained with hematoxylin and eosin and visualized by microscopy (Olympus). The ratio of neointimal hyperplasia was determined in hematoxylin and eosin‐stained paraffin‐embedded sections using ImageJ by assessing the neointimal to medial areas (N/M) under blinded conditions. The N/M from six to eight sections for each animal were pooled to generate the mean. The mean and standard error of the mean (SEM) was calculated from the collated data of each treatment group.

For immunofluorescence analysis, sections were dewaxed in xylene, then rehydrated in a stepwise manner in ethanol. The sections were treated with antigen retrieval for 10 minutes at 120°C, then normal goat serum for 30 minutes to reduce nonspecific binding. Sections were incubated with anti‐proliferating cell nuclear antigen (PCNA) antibody (Cell Signaling Technology, Beverly, MA, USA) overnight at 4°C, followed by FITC‐conjugated secondary antibodies (Invitrogen). Cell nuclei were counterstained with DAPI (Sigma‐Aldrich) and sections were visualized by fluorescence microscopy. The number of neointimal cells with DAPI‐stained nuclei and PCNA‐positive cells were counted after vascular ligation to determine the percentage of PCNA‐positive cells in the neointima. The mean for each animal was generated from five sections.

### 2.13 Statistical analysis

Data are expressed as the mean ± SD. Statistical significance was determined by an unpaired Student's *t* test or one‐way ANOVA with the Bonferroni post‐hoc test using GraphPad Prism 5.0 software. The results of all of the in vitro experiments are representative of at least three separate experiments conducted in triplicate and *P *<* *0.05 was considered to indicate a statistically significant difference.

## 3 RESULTS

### 3.1 MSC‐Exo reduce VSMC proliferation and migration

**Figure 1:**
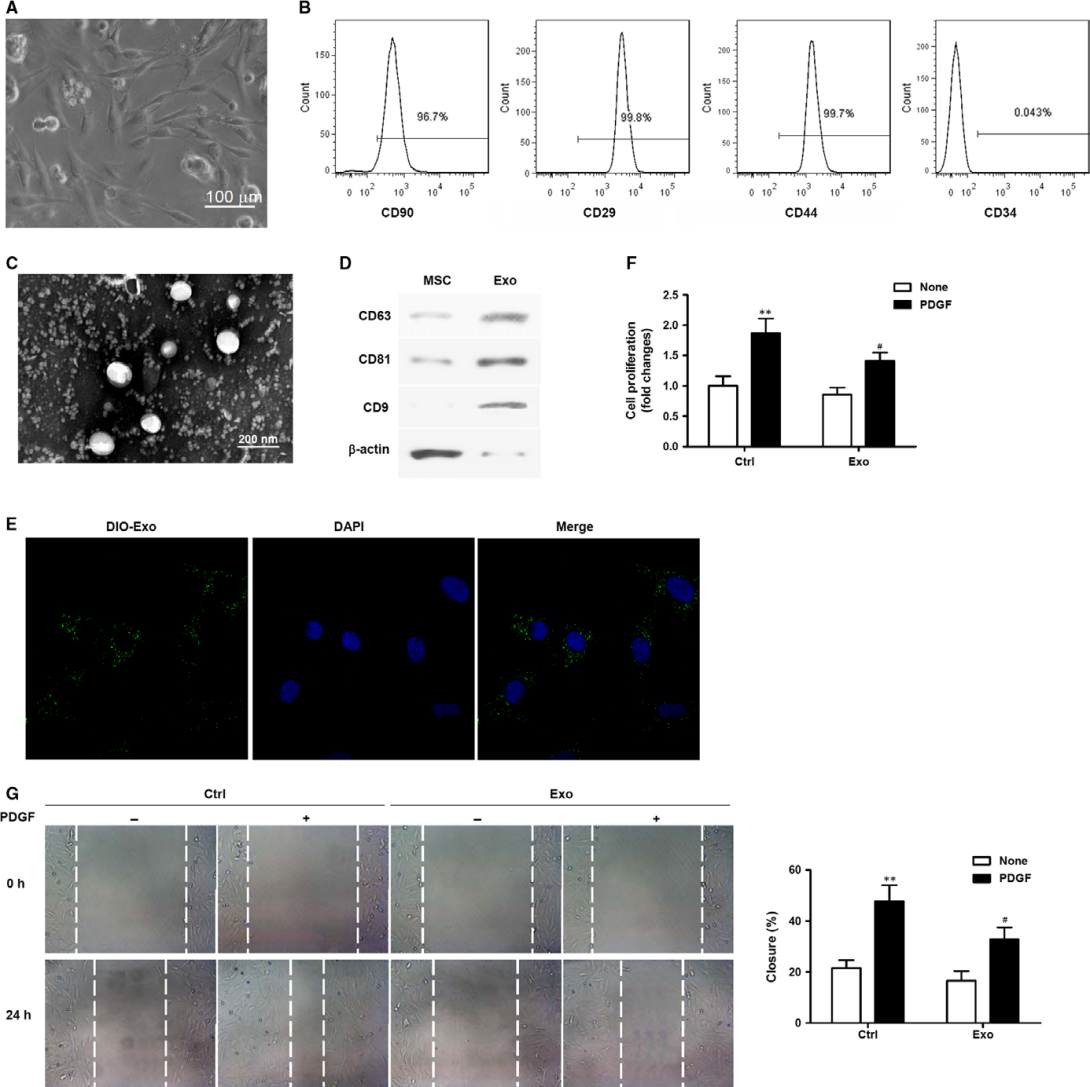
Mesenchymal stem cells (MSC)‐derived exosomes reduced proliferation and migration of vascular smooth muscle cells (VSMCs). (A) The morphology of MSCs was observed under a microscope; scale bar = 100 μm. (B) Representative fluorescence‐activated cell sorting (FACS) analysis of MSCs expressing antigens for CD90, CD29, CD44 and CD34. C. Micrographs obtained by transmission electron microscopy of MSC‐derived exosomes. (D) Western blot assays to detect exosome/extracellular vesicle‐specific markers (CD63, CD81, CD9) in MSCs and purified exosomes. (E) Representative micrographs obtained by confocal microscopy of DIO‐labelled exosome (green) uptake by VSMCs. Nuclear counterstaining was performed with DAPI (blue). (F) Analysis of VSMC proliferation by MTT assays. VSMCs were treated with or without MSC‐Exo in the presence or absence of PDGF‐BB (20 ng/mL) for 48 h. Three independent experiments (n* = *3). (G) Cell migration was measured after treatment with or without MSC‐Exo in the presence or absence of PDGF‐BB (20 ng/mL) over 24 h by scratch‐wound assays and is presented as the percentage of cell closure. Experiments were repeated at least three times (n* = *3) and data are the average of repeat experiments. **P <* *0.01 vs no PDGF treatment, ^#^ *P *<* *0.05 vs control cells

The establishment of an MSC culture was confirmed visually by the presence of elongated and linear cells and by flow cytometry with antibodies directed against CD90, CD29, CD44 and CD34 (Figure 1A,B). To evaluate the effects of MSC‐Exo on neointimal hyperplasia, MSC‐Exo were isolated and the morphology of the exosomes was observed using TEM. The exosomes purified from the MSC culture supernatants were round membrane‐bound vesicles that were ~50‐100 nm in diameter (Figure 1C). Then, Western blot analysis was performed on the proteins from MSC lysates and purified exosomes to confirm the presence of exosome/extracellular vesicle‐specific markers: CD63, CD81 and CD9. As expected, all three markers were strongly expressed in the purified exosomes (Figure 1D). Next, VSMCs were incubated with DIO‐labelled exosomes and confocal microscopy was performed to demonstrate the internalisation of DIO‐labelled exosomes (green) by VSMCs, whose nuclei were counterstained with DAPI (blue), and uptake of exosomes by VSMCs was observed (Figure 1C). To analyse the effect of MSC‐Exo on VSMC proliferation, an MTT assay was performed on VSMCs treated with or without MSC‐Exo in the presence or absence of PDGF‐BB (20 ng/mL) for 24 hours (Figure 1F). PDGF‐BB is a growth factor that stimulates intracellular signal transduction pathways and promotes VSMC proliferation and migration. Cell proliferation was significantly increased in the presence of PDGF‐BB (*P *<* *0.01 compared with untreated cells) but was lower in VSMCs treated with MSC‐Exo (*P *<* *0.05 compared with control cells) indicating that the presence of MSC‐Exo had a suppressive effect on VSMC proliferation. To analyse the effect of MSC‐Exo on VSMC cell migration, a scratch‐wound assay was performed. Similar to cell proliferation, cell migration was significantly increased in the presence of PDGF‐BB (*P *<* *0.01 compared with untreated cells) but was lower in VSMCs treated with MSC‐Exo (*P *<* *0.05 compared with control cells) (Figure 1G) indicating that the presence of MSC‐Exo also had a suppressive effect on VSMC migration.

### 3.2 MSC‐Exo deliver miR‐125b into VSMCs

**Figure 2:**
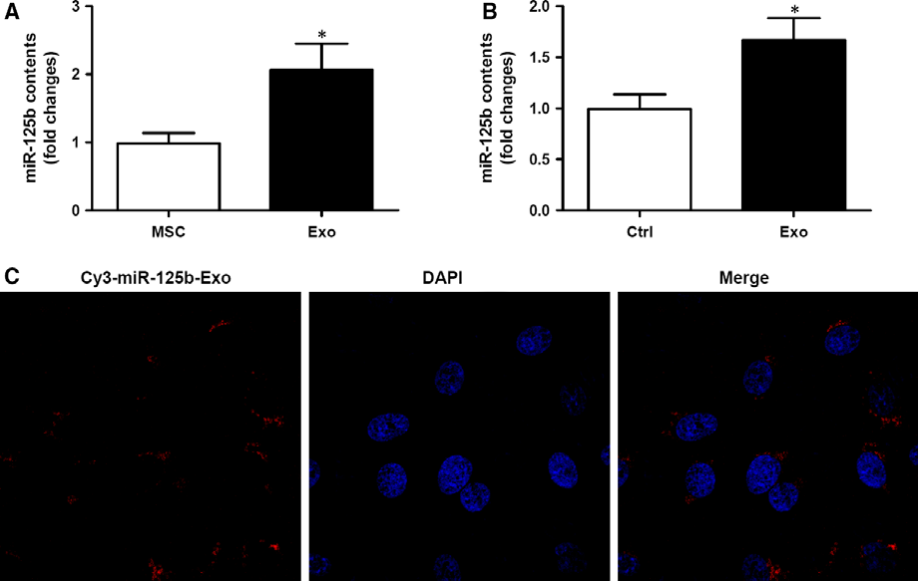
Mesenchymal stem cells (MSC)‐derived exosomes delivered miR‐125b into vascular smooth muscle cells (VSMCs). (A) Relative expression levels of miR‐125b in MSCs and MSC‐derived exosomes as determined by RT‐qPCR. Three independent experiments (n* = *3). (B) Expression levels of miR‐125b in VSMCs with and without treatment with exosomes as determined by RT‐qPCR. *P <* *0.05 vs untreated cells. Three independent experiments (n* = *3). (C) Exosomes were isolated from MSCs transfected with Cy3‐miR‐125b for 48 h and incubated with VSMCs for 3 h. Localisation of Cy3‐miR‐125b (red) in the exosome‐treated VSMCs was observed using a confocal microscope. Nuclear counterstaining was performed with DAPI (blue)

To investigate the MSC‐Exo‐mediated transfer of the miRNA, miR‐125b, into VSMCs, the relative expression levels of miR‐125b in MSCs and MSC‐Exo were first determined by quantitative reverse‐transcription PCR (RT‐qPCR). Expression levels of miR‐125b were significantly higher in MSC‐Exo than in MSCs (*P *<* *0.05; Figure 2A). Next, the expression of miR‐125b in VSMCs after incubation with MSC‐Exo was determined by RT‐qPCR and significantly higher miR‐125b expression was detected in VSMCs incubated with MSC‐Exo compared with control VSMCs (*P *<* *0.05; Figure 2B). To demonstrate the transfer of miRNA from MSCs into VSMCs via exosomes, exosomes were isolated from MSCs transfected with Cy3‐miR‐125b for 48 hours and were then incubated with VSMCs for 3 hours. Localisation of Cy3‐miR‐125b (red) in the exosome‐treated VSMCs (counterstained with DAPI, blue) was observed using a confocal microscope (Figure 2C). This provided evidence for the exosome‐mediated transfer of miR‐125b from MSCs into VSMCs.

### 3.3 miR‐125b delivered by MSC‐Exo inhibits VSMC proliferation and migration

**Figure 3:**
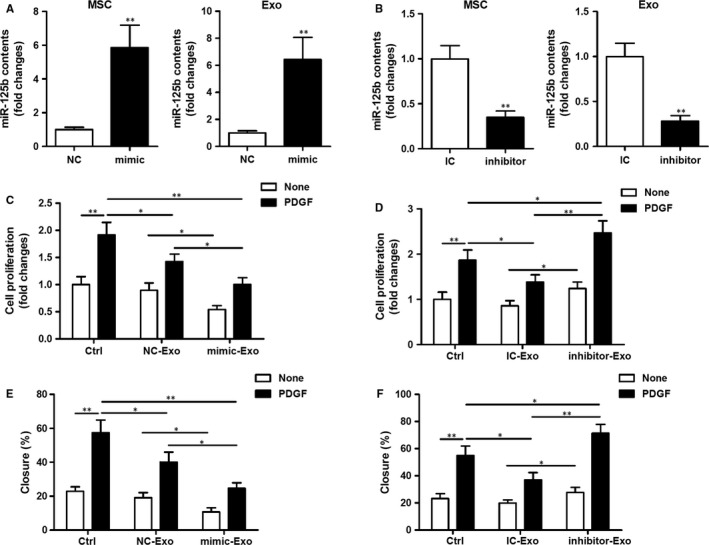
miR‐125b delivered by exosomes modulates vascular smooth muscle cells (VSMC) proliferation and migration. (A, B) Mesenchymal stem cells (MSCs) were transfected with 50 nmol/L miR‐125b mimic or mimic control (NC) (A), or 100 nmol/L inhibitor or inhibitor control (IC) (B) as indicated. miR‐125b expression was analysed in MSCs or in MSC‐derived exosomes by RT‐qPCR. **P <* *0.01 vs NC or IC. (C, D) Analysis of VSMC proliferation by MTT assays. VSMCs were treated with exosomes isolated from MSCs transfected with miR‐125b mimic (C) or inhibitor (D) in the presence or absence of PDGF‐BB (20 ng/mL) for 48 h and cell proliferation was determined. *P <* *0.05*,* **P <* *0.01. (E, F) Cell migration was measured after treatment with exosomes isolated from MSCs transfected with miR‐125b mimic (E) or inhibitor (F) in the presence or absence of PDGF‐BB (20 ng/mL) by scratch‐wound assays and is presented as the percentage of cell closure. Experiments were repeated at least three times (n = 3) and data are the average of repeat experiments. *P <* *0.05, **P <* *0.01

To investigate whether miR‐125b transferred by MSC‐Exo into VSMCs plays a role in the exosome‐mediated suppression of VSMC proliferation and migration, MSCs were transfected with miR‐125b mimic or mimic control (NC), or miR‐125b inhibitor or IC, and miR‐125b expression was analysed in MSCs and in MSC‐Exo (Figure 3A,B). In MSCs transfected with miR‐125b mimic, a significant increase in miR‐125b expression was detected in MSCs and in MSC‐Exo compared with the NC (*P *<* *0.01; Figure 3A) confirming the overexpression of this miRNA. By contrast, in MSCs transfected with miR‐125b inhibitor, a significant decrease in miR‐125b expression was detected in MSCs and in MSC‐Exo compared with the IC (*P *<* *0.01; Figure 3B) confirming effective inhibition of the expression of this miRNA.

Next, exosomes derived from these MSC strains were incubated with VSMCs to investigate the effects of exosomal‐transferred miR‐125b on VSMC proliferation and migration. VSMC proliferation was decreased following treatment with exosomes from MSCs transfected with miR‐125b mimic compared with exosomes from NC‐transfected MSCs (*P *<* *0.05; Figure 3C). Conversely, VSMC proliferation was increased following treatment with exosomes from MSCs transfected with miR‐125b inhibitor compared with exosomes from IC‐transfected MSCs (*P *<* *0.05; Figure 3D).

Next, the effects of MSC‐derived exosomally transferred miR‐125b on VSMC migration were assessed using the same MSC transfected strains and a scratch‐wound assay. VSMC migration was decreased following treatment with exosomes from MSCs transfected with miR‐125b mimic compared with exosomes from NC‐transfected MSCs (*P *<* *0.05; Figure 3E). Conversely, VSMC migration was increased following treatment with exosomes from MSCs transfected with miR‐125b inhibitor compared with exosomes from IC‐transfected MSCs (*P *<* *0.05; Figure 3F). Taken together, these findings indicate that miR‐125b delivered by MSC‐Exo inhibits VSMC proliferation and migration.

### 3.4 Exosomal miR‐125b suppresses VSMC proliferation and migration by targeting Myo1e

**Figure 4:**
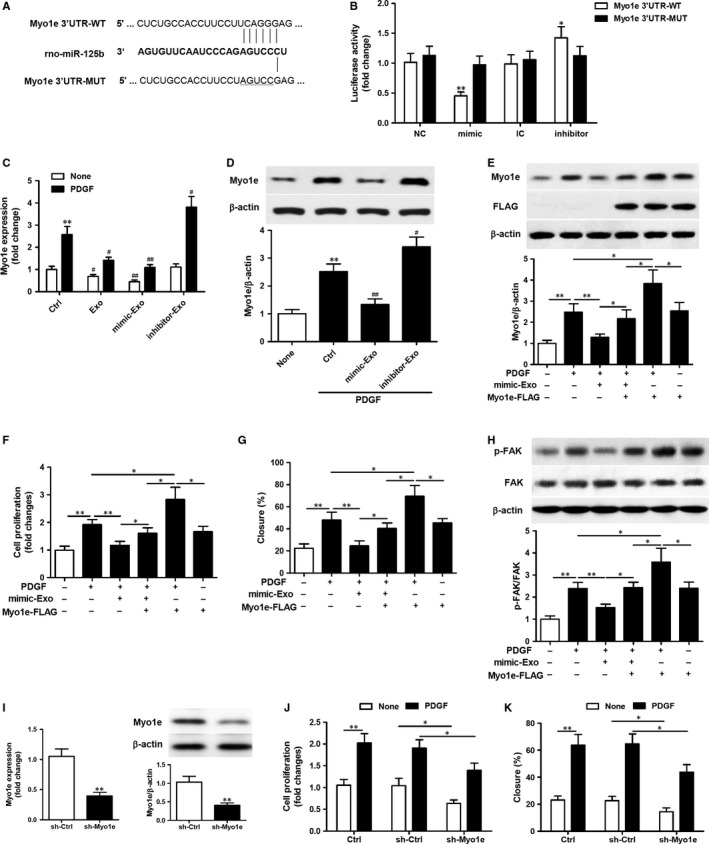
miR‐125b shuttled by mesenchymal stem cells (MSC)‐exosomes suppresses vascular smooth muscle cells (VSMC) proliferation and migration by targeting Myo1e. A. Schematic representation of the homologous sequence between rat Myo1e 3ʹUTR mRNA and miR‐125b. B. Dual luciferase assay on wild‐type (WT) or mutant (MUT) rat Myo1e 3ʹUTR in VSMCs transfected with miR‐125b mimic (or mimic control, NC) or inhibitor (or inhibitor control, IC). Data represent the luciferase activity ratio of firefly to Renilla luciferase. *P <* *0.05, **P <* *0.01 vs NC or IC. Three independent experiments (n* = *3). (C) RT‐qPCR analysis of the expression of Myo1e in VSMCs after treatment with exosomes isolated from MSCs or MSCs transfected with miR‐125b mimic or inhibitor in the presence or absence of PDGF‐BB (20 ng/mL) for 24 h. **P <* *0.01 vs no PDGF‐BB control; ^#^ *P *<* *0.05, ^##^ *P *<* *0.01 vs control cells. Three independent experiments (n* = *3). (D) Western blot analysis of Myo1e in VSMCs after treatment with exosomes isolated from MSCs transfected with miR‐125b mimic or inhibitor in the presence of PDGF‐BB (20 ng/mL) for 24 h. **P <* *0.01 vs no PDGF‐BB control; ^#^ *P *<* *0.05, ^##^ *P *<* *0.01 vs control cells. β‐actin was detected as a loading control. Three independent experiments (n = 3). (E‐H) VSMCs transfected with Myo1e overexpression plasmid for 24 h were stimulated with or without exosomes isolated from MSCs transfected with miR‐125b mimic in the presence of PDGF‐BB (20 ng/mL). (E) Western blot analysis of Myo1e expression in the treated VSMCs. (F) Analysis of VSMC proliferation by MTT assays. (G) The percentage of cell closure as measured by scratch‐wound assays. (H) Western blot analysis to examine FAK phosphorylation. β‐actin was detected as a loading control. (I‐K) VSMCs transfected with lentiviral particles of the Myo1e shRNA and lenti control shRNA for 48 h, stimulated with or without PDGF‐BB (20 ng/mL) for 24 h. (I) RT‐qPCR analysis and Western blot analysis of Myo1e expression in the VSMCs. (J) Analysis of VSMC proliferation by MTT assays. (K) The percentage of cell closure as measured by scratch‐wound assays. *P <* *0.05, **P <* *0.01. Three independent experiments (n* = *3)

Next, the mechanism responsible for exosomal miR‐125b‐mediated suppression of VSMC proliferation and migration was investigated. A miR‐125b binding site was identified in the 3ʹUTR of rat Myo1e (Figure 4A). Myo1e is an actin‐dependent molecular motor previously reported to play a role in lamellipodium extension and subsequent cell migration.^26^ A dual luciferase assay was therefore performed with WT and mutant (MUT) constructs of the Myo1e 3′UTR in VSMCs transfected with miR‐125b mimic or inhibitor (Figure 4B). Transcription of the 3ʹUTR of WT Myo1e, as indicated by luciferase activity, was significantly decreased in VSMCs transfected with miR‐125b mimic compared with the NC (*P *<* *0.01), and significantly increased in VSMCs transfected with miR‐125b inhibitor compared with the IC (*P *<* *0.05). These effects were abolished when the MUT 3ʹUTR construct was used, indicating the specific interaction between miR‐125b and the 3ʹUTR of rat Myo1e.

To further confirm the effect of miR‐125b on Myo1e expression, RT‐qPCR analysis of Myo1e expression was performed on VSMCs (Figure 4C). As expected, in the presence of PDGF‐BB, Myo1e expression was decreased in MSC‐Exo‐treated VSMCs (*P *<* *0.05), further decreased in VSMCs treated with exosomes isolated from MSCs transfected with miR‐125b mimic (*P *<* *0.01) and increased in VSMCs treated with exosomes isolated from MSCs transfected with miR‐125b inhibitor (*P *<* *0.05) compared with control cells. In the absence of PDGF‐BB, the expression of Myo1e was reduced in control cells and the effects on Myo1e expression in treated cells followed a similar pattern but were far less pronounced. These results were consistent with the Myo1e protein expression levels (Figure 4D). Myo1e protein expression was decreased in VSMCs treated with exosomes isolated from MSCs transfected with miR‐125b mimic (*P *<* *0.01) and increased in VSMCs treated with exosomes isolated from MSCs transfected with miR‐125b inhibitor (*P *<* *0.05) compared with control cells.

Next, a Myo1e overexpression plasmid was used to transfect VSMCs and then VSMCs overexpressing Myo1e were stimulated with or without exosomes isolated from MSCs transfected with miR‐125b mimic in the presence of PDGF‐BB (20 ng/mL) for 24 hours. Western blot analysis confirmed the overexpression of Myo1e in VSMCs transfected with the Myo1e overexpression plasmid in the absence of exosome treatment. In the presence of exosomes isolated from MSCs transfected with miR‐125b mimic, Myo1e expression was decreased (*P *<* *0.05; Figure 4E). VSMCs overexpressing Myo1e were then subjected to functional assays including a cell proliferation assay and a scratch‐wound assay to measure cell migration. Myo1e overexpression restored the inhibition of VSMC proliferation (Figure 4F) and migration (Figure 4G) in response to treatment with exosomes isolated from MSCs transfected with miR‐125b mimic.

Focal adhesion kinase has been reported to play a role in the proliferation and migration of aortic smooth muscle cells, and the regulation of smooth muscle cell recruitment during blood vessel morphogenesis.^28^, ^29^, ^30^ Myo1e has previously been reported to induce FAK phosphorylation and FAK kinase activity.^27^ Here, the phosphorylation of FAK was examined by Western blot analysis in the VSMCs after the indicated treatment (Figure 4H). FAK phosphorylation was promoted by the overexpression of Myo1e but decreased by treatment with exosomes isolated from MSCs transfected with miR‐125b mimic. Transfection of VSMCs with lentiviral particles of Myo1e shRNA reduced the expression of Myo1e mRNA and the levels of protein (Figure 4I). Moreover, cell proliferation and migration were both reduced in VSMCs with silenced Myo1e expression (Figure 4J,K). Stimulation by PDGF‐BB led to increased levels of cell proliferation and migration in the control VSMCs but these levels were significantly lowered in VSMCs with Myo1e silenced (Figure 4J,K). Taken together, these findings indicate that exosomal miR‐125b suppresses VSMC proliferation and migration by targeting Myo1e and the subsequent FAK activation.

### 3.5 miR‐125b delivered by MSC‐Exo reduces neointima formation in rat carotid arteries after angioplasty

**Figure 5:**
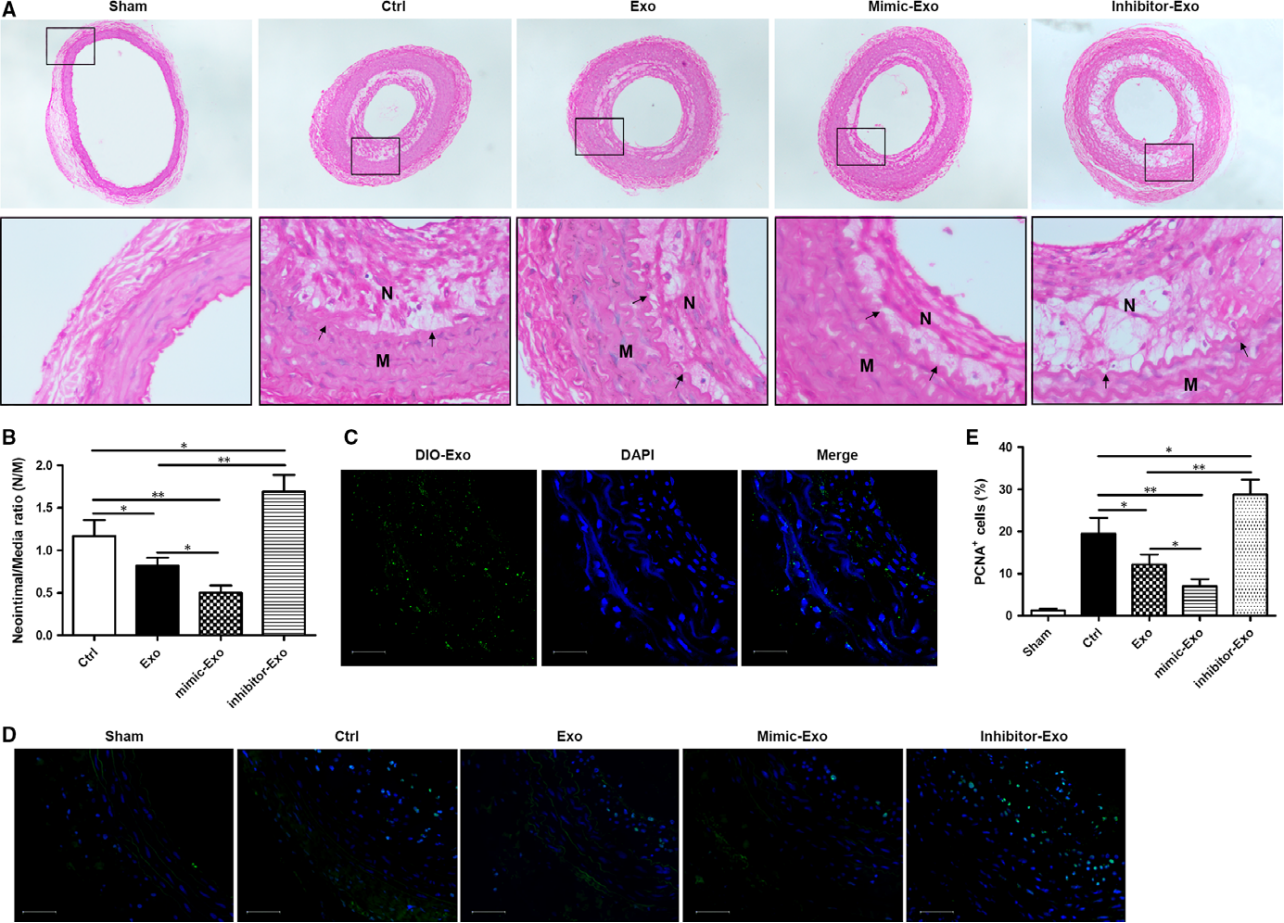
miR‐125b delivered by mesenchymal stem cells (MSC)‐exosomes attenuates neointimal formation in a rat carotid artery balloon injury model. (A) Representative images of hematoxylin and eosin immunostaining of rat aortic sections after carotid artery balloon injury and intravenous injection of exosomes derived from untransfected MSCs or MSCs transfected with miR‐125b mimic or inhibitor. Arrows indicate the neointima (N) and media (M). (B) Quantification of the neointima/media ratio in the injured carotid arteries from the different groups. The neointima/media ratio is expressed as the mean ± SEM (n = 6 in each group). (C) Analysis by immunofluorescence of exosome distribution (green) in rat carotid arteries 2 days after the last time of intravenous injection of DIO‐labelled exosomes. Nuclear counterstaining was performed with DAPI (blue). Scale bar = 50 μm. (D) Representative immunofluorescence images of PCNA (green) in rat carotid arteries 14 days after balloon injury and intravenous injection of exosomes derived from untransfected MSCs or MSCs transfected with miR‐125b mimic or inhibitor. Nuclear counterstaining was performed with DAPI (blue). Scale bar = 50 μm. (E) Quantification of the percentage of PCNA‐positive cells within the neointima of different groups following carotid ligation. Data are expressed as the mean ± SEM. n = 4‐5. *P <* *0.05, **P <* *0.01 vs untreated control cells

To analyse the effects of exosome‐derived miR‐125b on neointima formation in rat carotid arteries after angioplasty, a rat model of balloon‐induced vascular injury was employed. Following balloon injury, 20 μg of exosomes derived from untransfected MSCs or MSCs transfected with miR‐125b mimic or inhibitor was injected intravenously over a 2‐week period. The left common carotid artery was harvested, sectioned and immunostained with hematoxylin and eosin (Figure 5A). The N/M of the injured carotid arteries was determined for each treatment group (Figure 5B). Analysis by immunofluorescence of DIO‐labelled exosomes clearly indicates the distribution of MSC‐Exo in rat carotid arteries (Figure 5C). It was evident that the delivery of miR‐125b by MSC‐Exo reduced neointima formation, whereas the delivery of MSC‐Exo in which miR‐125b was inhibited increased neointima formation in rat carotid arteries following angioplasty.

Proliferating cell nuclear antigen, which is detectable by immunofluorescence, is positively correlated with the proliferative state of cells. PCNA immunofluorescence in rat carotid arteries at 14 days after balloon injury was detected (Figure 5D), and the percentage of PCNA‐positive cells within the neointima of the different groups after carotid ligation was determined (Figure 5E). It was evident that the delivery of miR‐125b by MSC‐Exo reduced cell proliferation within the neointima, whereas the delivery of MSC‐Exo in which miR‐125b was inhibited decreased proliferation of cells in rat carotid arteries following angioplasty. Taken together, these findings confirmed the important role of MSC‐Exo‐derived miR‐125b in reducing neointima formation in rat carotid arteries following vascular injury.

## 4 DISCUSSION

All types of surgical coronary intervention result in some degree of arterial injury, the response to which determines the success of the procedure and the long‐term prognosis of the patient. Stent implantation, for example, promotes neointimal hyperplasia, a major cause of in‐stent restenosis.^3^, ^4^, ^5^, ^6^ Current strategies to prevent restenosis are focused on the inhibition of neointimal hyperplasia through drug‐eluting stents and vascular brachytherapy. It is, therefore, crucial to understand the molecular mechanisms involved in neointima formation.

Intimal hyperplasia is the thickening of a blood vessel wall in response to injury and involves the proliferation and migration of VSMCs and the deposition of extracellular matrix. When this process of rapid re‐endothelialisation occurs on a prosthesis, the new layer of arterial intima is referred to as the neointima. VSMC proliferation and subsequent migration into the neointima layer following injury have been shown to be promoted by PDGF‐BB,^35^ but the molecular mechanism involved remained to be fully elucidated. It has previously been established that MSCs affect VSMCs via exosome‐mediated intercellular communication between these cells.^9^ Furthermore, it was reported that the exosome‐mediated transfer of biological material and signalling molecules (such as mRNA and miRNA) between these cell types could induce altered behaviour in the recipient cells, such as triggering angiogenesis.^13^, ^36^, ^37^ Similarly, the exosome‐mediated transfer of miRNAs from MSCs to endothelial cells has been reported to promote angiogenesis.^38^

Extracellular vesicles, of which exosomes are the smallest in size (~30‐100 nm in diameter), contain proteins, lipids, miRNAs and other noncoding RNAs. It is via the transfer of this protein, lipid and nucleic acid content to target cells that extracellular vesicles exert remote functional effects. Increased levels of circulating extracellular vesicles have been observed during cardiovascular diseases, including myocardial infarction, and there is also evidence for the role of exosomes in atherosclerosis, neointima formation and vascular repair and remodelling. However, to date, research into the role of extracellular vesicles in disease has relied on vesicles isolated from cultured cells and may not fully reflect the in vivo situation. For example, exosomes derived from endothelial progenitor cells (EPCs) were used to study neointima formation and vascular repair in a rat model of balloon‐induced vascular injury and revealed attenuated vascular repair, enhanced re‐endothelialisation in vivo and endothelial function in vitro.^39^ Similarly, a study into the role of exosomes in pulmonary arterial hypertension employed MSC‐Exo and revealed their cytoprotective effects during pulmonary hypertension, and subsequently demonstrated that intravenous delivery of MSC‐Exo inhibited vascular remodelling.^40^ In another study, MSC‐Exo were shown to exert anti‐inflammatory effects on macrophages and aortic smooth muscle cells, reducing the incidence of aortic aneurysm.^41^

The migration and proliferation of smooth muscle cells are important in the development of atherosclerosis and extracellular vesicles secreted from a range of cell types may affect these processes, specifically via exosomal miRNAs.^42^, ^43^ Shan et al (2015) demonstrated that extracellular vesicle‐derived miR‐223 inhibited VSMC migration and proliferation, resulting in a reduction in the size of atherosclerotic plaques. Similarly, extracellular vesicle‐derived miR−143 and miR−145 were shown to inhibit the dedifferentiation of VSMCs.^43^ Extracellular vesicles derived from adipose MSCs have been shown to limit neointimal hyperplasia and in a study by Liu et al (2015)^44^ this was attributed to decreased macrophage infiltration, reduced expression of inflammatory cytokines and downregulation of the MAPK and PI3K signalling pathways. In another study, exosomes derived from pulmonary artery endothelial cells were shown to induce high levels of proliferation and exert anti‐apoptotic effects in pulmonary artery smooth muscle cells, contributing to vascular remodelling and the pathogenesis of pulmonary hypertension.^45^

Accumulating evidence suggests that exosome‐derived miRNAs may be involved in the regulation of various aspects of neointima formation via the modulation of target cell functions (eg proliferation and migration of VSMCs) and the resulting effects on vasculature homeostasis.^39^, ^46^, ^47^, ^48^ It has been reported that MSC engraftment reduces vascular remodelling after coronary vessel balloon injury, this was demonstrated by a lower I/M, neointimal area and positive expression of PCNA in addition to enhanced re‐endothelialisation after MSC transplantation.^46^, ^47^ Several studies have reported that exosomes derived from stem cells could target endothelial cells to promote the pro‐angiogenic effect after myocardial infarction, and exosomes derived from MSC could target VSMC to promote an anti‐inflammatory effect.^48^ In a rat model of balloon‐induced vascular injury, exosomes isolated from EPCs accelerated the process of re‐endothelialisation in vivo and enhanced endothelial function in vitro.^39^ Exosomes secreted by MSCs have been shown to mediate intercellular communications between these cells and their target cells; however, the role of MSC‐Exo in neointimal hyperplasia remains to be fully elucidated.

Following arterial injury resulting from coronary interventions, miRNAs can selectively inhibit smooth muscle cell proliferation, promote re‐endothelialisation and inhibit platelet activation indicating their potential as therapeutic candidates. Various studies have been reported demonstrating the functional role of particular exosomal miRNAs. Gu et al (2017)^49^ reported that extracellular vesicles secreted from endothelial cells transferred miR‐195 to smooth muscle cells in vessels where this miRNA inhibited cellular proliferation, thereby protecting against vessel restenosis. Similarly, Deng et al (2015)^50^ demonstrated that miR‐143‐3p exosomally transferred from pulmonary artery smooth muscle cells exerted a paracrine pro‐migratory and pro‐angiogenic effect on pulmonary arterial endothelial cells. In another study, miR‐125a‐5p, which was downregulated following vascular injury, was shown to modulate phenotypic switching of VSMCs, a key event in the development of restenosis following coronary intervention. miR‐125a‐5p had a direct effect on VSMC proliferation and migration.

Several studies have recognized miR‐125b as an important mediator for the modulation of the SMC phenotype.^23^, ^25^, ^51^ Deregulation of VSMC phenotype switching is associated with vascular proliferative and migration disorders.^52^ Through literature searches, we found that miR‐125b was enriched in MSC‐Exo,^53^, ^54^ therefore, we investigated the role of MSC‐Exo‐mediated miR‐125b transfer on neointimal hyperplasia following arterial injury. The findings from our study indicated that MSC‐Exo are enriched in miR‐125b and can transfer this miRNA to VSMCs. In previous studies, miR‐125b was reported to play a role in osteoblast differentiation by regulating cell proliferation,^23^ and osteogenic transdifferentiation of VSMCs and the proliferation of human coronary artery smooth muscle cells.^25^ In our study, we demonstrated that miR‐125b targets the 3ʹUTR of Myo1e in VSMCs repressing its expression and affecting subsequent FAK activation. The interaction between Myo1e and FAK and the resulting phosphorylation‐activation of FAK has been shown to be crucial for cell motility,^27^, ^28^ and therefore the transcriptional repression of Myo1e would be expected to inhibit the migratory behaviour of VSMCs.

Myosin 1E is an actin‐dependent molecular motor that is expressed in a range of tissues and has the ability to bind to both cell membranes and actin filaments. Myo1e also binds phospholipids with high affinity^55^ and regulates podocyte function and glomerular filtration.^56^ Furthermore, the ERK‐mediated translocation of Myo1e from the cytosol to the tips of lamellipodia is essential for cell motility and migration 57 and Myo1e regulates TLR4‐triggered macrophage spreading and chemokine release as part of the innate immune response.^58^ Myo1e has therefore been linked to a number of diseases such as atherosclerosis,^59^ coronary artery disease and kidney disease.^60^

In our study, using a rat model of balloon‐induced vascular injury, we revealed that exosome‐derived miR‐125b repressed Myo1e expression, suppressed VSMC proliferation and migration and inhibited neointima formation in rat carotid arteries following angioplasty. Our findings suggest that miR‐125b may be a potential therapeutic candidate for the treatment of vascular diseases and arterial injury. It is conceivable that this miRNA could be delivered to the site of injury by incorporation into a drug‐eluting stent.

## CONFLICTS OF INTEREST

The authors confirm that there are no conflicts of interest.

## AUTHORS’ CONTRIBUTIONS

D.Q.W. and B.G. performed experiments, analysed the data and contributed to the preparation of the manuscript. J.N.Y., F.L., and Y.F.L. performed experiments. W.G.F. and Y.S. designed the research study, analysed the data and wrote the manuscript.
